# Combination of Preoperative D-Dimer and Platelet Distribution width Predicts Postoperative Deep Venous Thrombosis in Patients with Cervical Carcinoma

**DOI:** 10.31557/APJCP.2019.20.4.1025

**Published:** 2019

**Authors:** Na Li, Fu-Bin Zhang, Bing-Jie Li, Rui-Tao Wang

**Affiliations:** 1 *Department of Internal Medicine,*; 2 *Department of Gynecology, *; 3 *Department of Ultrasound, Harbin Medical University Cancer Hospital, Harbin Medical University, Harbin, Heilongjiang, China.*

**Keywords:** Cervical carcinoma, D-dimer, activated platelets, platelet distribution width, risk prediction

## Abstract

**Background:**

Deep venous thrombosis (DVT) is associated with severe morbidity and mortality in cancer. Platelet distribution width (PDW), a platelet index, indicates variation in platelet size. We aimed to investigate whether the combination of D-dimer and PDW could have a better performance in predicting DVT in patients with cervical carcinoma.

**Materials and Methods:**

In 198 consecutive cervical carcinoma patients without preoperative DVT, preoperative D-dimer and PDW levels were measured. Compression ultrasonography was performed in all cervical carcinoma patients before surgery, as well as one month, three months, six months, and 12 months.

**Results:**

During a median period of 12 months, 17 of the 198 patients (8.6 %) developed DVT. PDW levels were reduced and D-dimer levels were increased in patients with DVT events compared to those without DVT. Multivariate Cox analysis revealed that both PDW and D-dimer were independent predictors for DVT events. The area under the ROC curve was 0.628 (95% CI: 0.556 to 0.695, p=0.142) when D-dimer was used alone, whereas it increased to 0.777 (95% CI: 0.712 to 0.833, p<0.011) with the addition of PDW. Incorporation of PDW into the D-dimer model significantly improved the predictive value.

**Conclusions:**

The combination of preoperative D-dimer and PDW improves the predictive power of postoperative DVT risk in patients with cervical carcinoma.

## Introduction

Deep venous thrombosis (DVT) is associated with severe morbidity and mortality in cancer. Fifty percent of DVTs are asymptomatic and 30% will have additional complications (Heit et al.,1999). Therefore, accurate prediction for DVT is of utmost clinical importance. D-dimer, a fibrin degradation product, is the most well established biomarker used to assesss for DVT in clinical practice. However, there are nonspecific D-dimer elevations in cancer patients. 

Cervical cancer is the most common malignant tumor of the female reproductive system in China. Despite the availability of early screening programs, the morbidity of cervical cancer remains high.

Platelets play a key role in atherogenesis, inflammation, and atherothrombosis (Linden and Jackson, 2010). Platelet distribution width (PDW), a platelet index, indicates variation in platelet size and differentially diagnoses thrombocytopenia (Kaito et al., 2005). Accumulating evidence recently demonstrated that PDW is associated with poor prognosis in non-small cell lung cancer, gastric cancer, and laryngeal cancer (Cui et al., 2017; Zhang et al., 2017). 

In the present study, we aimed to investigate whether a combination of PDW and D-dimer prior to surgery could better predict the postoperative DVT in cervical carcinoma.

## Materials and Methods

*Study population*

The present study enrolled 277 consecutive patients who had been diagnosed with cervical carcinoma between 2014 and 2015 at the Harbin Medical University Cancer Hospital, China. All patients undergone complete surgical resection and all diagnoses were based on pathological evidence. None of the patients received preoperative chemotherapy or radiation therapy. The patients with the following characteristics were excluded from the present study: patients who had any coexisting or previous cancers other than cervical carcinoma; patients with concomitant diseases, including hematological disorders, hypertension, diabetes mellitus, atrial fibrillation, and patients who had taken anticoagulant or acetylsalicylic acid drugs in three months. 

Our study is a prospective study. A total of 277 patients enrolled in this study. 9 patients were excluded because they refused to give informed consent. 32 patients with hypertension, 38 patients with diabetes mellitus were also excluded. The final analysis included 198 patients. All subjects were followed–up for 12 months. This study complied with the Helsinki Declaration and was approved by the Human Ethics and Research Ethics committees of Harbin Medical University Cancer Hospital. Written informed consent was obtained from all patients.

**Table 1:** Baseline Characteristics of Patients According to the Postoperative DVT Status

Variables	With DVT	Without DVT	p value
Age (years)	56.8±10.0	46.9±7.4	< 0.001
BMI (kg/m^2^)	23.9±3.7	23.4±2.7	0.534
Smoker (n, %)	2 (11.8)	12 (6.6)	0.430
WBC (×10^9^/L)	6.90±2.81	5.84±1.77	0.144
Hemoglobin (g/dl)	117.8±20.5	124.0±13.6	0.088
Platelet count (×10^9^/L)	235.7±95.4	234.3±61.5	0.928
MPV (fL)	8.4±1.2	8.6±1.3	0.417
PDW (%)	16.1±2.4	17.2±1.1	0.001
D-dimer (µg/mL)	2.00±3.23	0.60±0.25	< 0.001
Operation time (minutes)	159.4±28.4	162.6±25.5	0.626
Postoperative fluid intake (ml/day)	1908.8±209.3	1873.8±199.3	0.491
Postoperative urine output (ml/day)	1873.5±271.6	1926.8±342.6	0.534
Duration of postoperative			
immobilization (days)	6.4±3.7	3.8±1.6	0.011
Differentiation			0.711
Well/moderate	11 (64.7)	125 (69.1)	
Poor	6 (35.3)	56 (30.9)	
Histological type			0.199
Adenocarcinoma	3 (17.6)	15 (8.3)	
Squamous cell carcinoma/others	14 (82.4)	166 (91.7)	
FIGO stage			0.046
I	5 (29.4)	99 (54.7)	
II	12 (70.6)	82 (45.3)	
Past history of VTE (n, %)	1 (5.9)	0 (0)	0.001

Data are expressed as means (SD) or median (IQR). DVT, deep venous thrombosis; WBC, white blood cell; BMI, body mass index; MPV, mean platelet volume; PDW, platelet distribution width; VTE, venous thrombo-embolism

**Table 2:** Univariate Cox Analysis

	Hazard ratio	95%CI	P-value
Age (years)	1.135	1.079–1.194	<0.001
BMI (kg/m^2^)	1.060	0.895–1.256	0.500
Smoker (n,%)	1.833	0.419–8.014	0.421
WBC (×10^9^/L)	1.262	1.038–1.536	0.020
Hemoglobin (g/dl)	0.973	0.944–1.003	0.080
Platelet count (×10^9^/L)	1.000	0.993–1.008	0.903
MPV (fL)	0.837	0.554–1.266	0.400
PDW (%) (≤15.8versus>15.8)	5.869	2.065–16.681	0.001
Operation time (minutes)	0.995	0.976–1.014	0.597
Postoperative fluid intake (ml/day)	1.001	0.998–1.003	0.458
Postoperative urine output (ml/day)	1.000	0.998–1.001	0.547
Duration of postoperative			
immobilization (days)	1.314	1.170–1.475	<0.001
D-dimer (µg/mL)			
(>0.94versus≤0.94)	4.793	1.824–12.597	0.001
Differentiation			
(Poor versus Well/moderate)	1.199	0.443–3.242	0.721
Histological type	1.289	0.460–3.616	0.629
FIGO stage			
II versus I	2.809	0.989–7.975	0.052
Past history of VTE (n,%)	24.247	3.033–193.862	0.003

Abbreviations: see to Table 1

**Table 3:** Multivariate Cox Proportional Hazards Analysis

	Hazard ratio	95%CI	P-value
Age (years)	1.118	1.044–1.197	0.001
WBC (×10^9^/L)	1.181	0.907–1.536	0.217
Hemoglobin (g/dl)	1.005	0.973–1.038	0.761
PDW (%)			
(≤ 15.8 versus > 15.8)	5.611	1.019–30.894	0.038
D-dimer (µg/mL)			
(> 0.94 versus ≤ 0.94)	9.707	2.611–36.080	0.001
FIGO stage			
(II versus I)	2.507	0.763–8.245	0.130
Past history of VTE (n, %)	1.026	0.096–10.933	0.983
Duration of postoperative			
immobilization (days)	1.309	1.133–1.512	< 0.001

Variables that showed a p-value < 0.10 in univariate analysis were included in a multivariate Cox proportional hazards regression model. CI, confidence interval. Abbreviations: see to Table 1.

**Figure 1:**
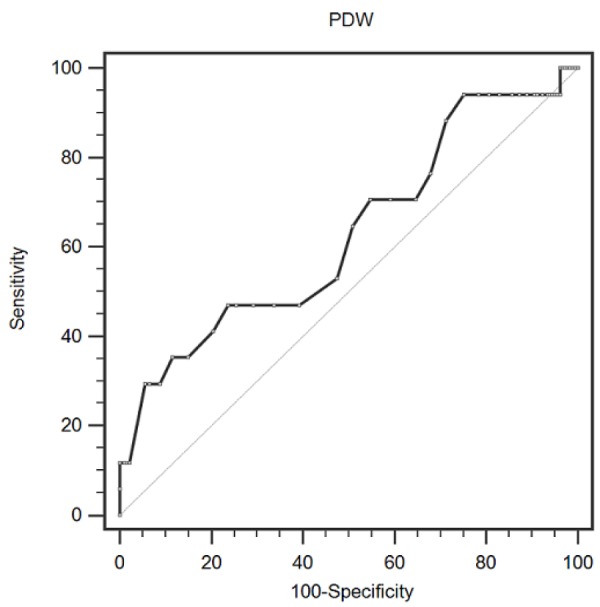
Optimized Cut-Off was Determined for PDW Using Standard ROC Curve Analysis

**Figure 2:**
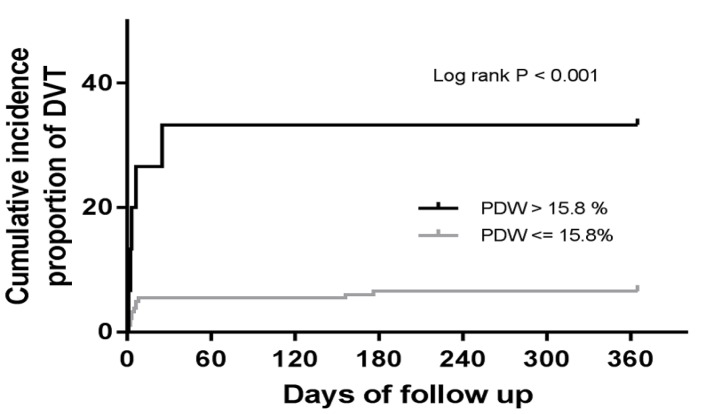
Kaplan–Meier Analysis of the Cumulative Incidence of DVT According to Preoperative PDW Levels

**Figure 3:**
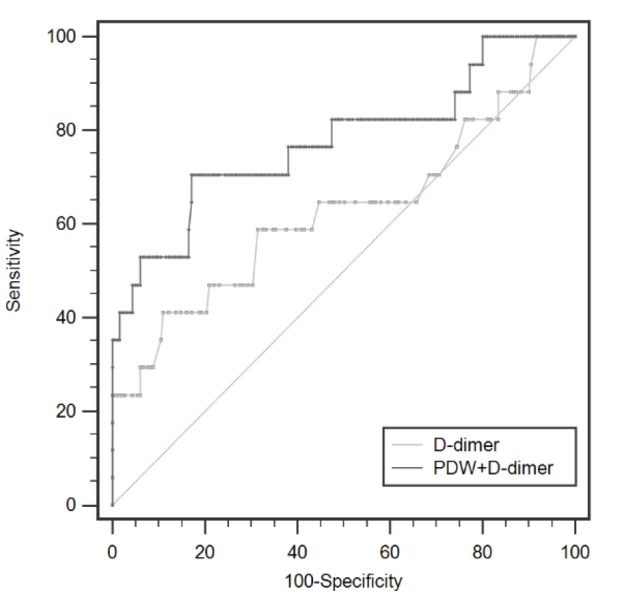
The Combination of PDW and D-Dimer Could Improve the Predictive Power of Postoperative DVT Using Standard ROC Curve Analysis

*Biochemical parameters*

All participants completed a standardized questionnaire including medical history, lifestyle factors and smoking habits. After a 10-hour overnight fast, blood samples were collected from participants. Platelet-poor plasma was then assayed using second-generation latex immunoassay kit that provides quantitative measure of D-dimer. It was analysed on Sysmex CS-5100 (Siemens Diagnostics, Erlangen, Germany). The standard cut-off value of D-dimer in our hospital is 0.55 mg/L. Platelet count and PDW levels were determined with an autoanalyzer (Sysmex XE-2100, Kobe, Japan). The whole blood samples were collected in EDTA-containing tubes and all samples were processed within 30 minutes after blood collection in order to prevent in vitro platelet activation. 

*Diagnosis of deep venous thrombosis*

An experienced ultrasonographist performed the compression ultrasonography according to standard procedures (grey scale, B-mode, color Doppler) with a high-end ultrasound scanner (DC-8 EXP, Mindray, Shenzhen, China) before surgery, as well as one month, three months, and six months. If the patients were in cases of clinical signs of DVT, an additional compression ultrasonography was performed. 

*Statistical analysis*

All statistical analyses were performed using SPSS Statistics version 22.0 (SPSS Inc., Chicago, IL, USA). Values for continuous variables are given as means ± SD or median (range). Values for categorical data are specified as frequency (percent). Student t test was used for the continuous variables, while the Chi-square test was used for the categorical variables. The Kaplan-Meier method was used to obtain the cumulative incidence proportion of DVT while statistical significance was compared between groups using the log-rank test. Uni- and multivariate analyses were performed using Cox’s proportional hazards regression model. Hazard ratios (HRs) estimated from the Cox-regression analysis were reported as relative risks with corresponding 95% confidence intervals. Receiver operating characteristic (ROC) curves were constructed for quantitative variables to determine the best cut-off point values. The area under the ROC curve (AUC) was calculated for the D-dimer model and for the combination of D-dimer and PDW. The differences in AUC were detected by using MedCalc version 15.0. For all analyses, a two-sided P-value of 0.05 or less was considered statistically significant. 

## Results

Data from the 198 remaining patients were analyzed. 17 (8.6 %) patients had death events. The baseline characteristics of the patients with DVT or without DVT events are summarized in Table 1. Compared to the patients without DVT, those with DVT were older, had a longer duration of postoperative immobilization, and higher frequency of venous thrombo-embolism. Additionally, PDW levels were reduced and D-dimer levels were increased in DVT patients.

A ROC curve for DVT prediction was plotted to verify the optimal cut-off value for PDW, which was 15.8 % (Figure 1). It demonstrated that PDW predicts DVT with a sensitivity of 29.4 % and a specificity of 94.5 % (AUC = 0.629, 95 % CI: 0.558-0.696). 

In the univariate Cox regression model, PDW, D-dimer, duration of postoperative immobilization, and past history of venous thrombo-embolism showed significant associations with a higher risk of DVT (Table 2). The multivariate Cox regression analysis revealed that PDW was an independent predictor for poor prognosis in patients with cervical carcinoma (HR, 4.945; 95% CI, 1.113–21.978; p = 0.036) (Table 3). 

The estimated cumulative incidence of DVT events by the Kaplan-Meier curve was shown in Figure 2. Patients with PDW ≤ 15.8 % had increased cumulative incidence of DVT compared with the patients with PDW > 15.8 % (33.3% vs. 6.6%, p < 0.001). 

As shown in Table 3, D-dimer could independently predict future DVT events in cervical carcinoma patients. To evaluate whether the combination of PDW and D-dimer could improve the prediction of DVT, ROC analysis was performed (Fig 3). The area under the ROC curve was 0.628 (95% CI: 0.556 to 0.695, p = 0.142) when D-dimer was used alone, whereas it increased to 0.777 (95% CI 0.712 to 0.833, p < 0.001) when the combination of PDW with D-dimer was used.

## Discussion

In this study, we observed that preoperative PDW has independent prognostic value for future postoperative DVT in patients with cervical carcinoma. In addition, the combination of D-dimer and PDW has a better performance in predicting DVT.

An increased preoperative D-dimer was associated with a higher cumulated incidence of postoperative deep venous thrombosis in patients with colorectal cancer (Stender et al., 2009). However, the nonspecific elevations of D-dimer in cancer patients limit its wider applicability (Schaefer et al., 2017). Our results showed that PDW confers an important additive effect to D-dimer. One possible explanation is that reduced PDW indicates platelet activation which exerts extensive effects on atherogenesis, inflammation, and atherothrombosis (Linden and Jackson, 2010). Bone marrow cells (including megakaryocytes) dys-regulation may contribute to changed PDW. PDW is a measure of platelet heterogeneity caused by heterogeneous demarcation of megakarocytes (Paulus,1981). Megakaryocytic maturation, platelet production and platelet size could be regulated by cytokines, such as interleukin-6 (IL-6), granulocytes colony stimulating factor (G-CSF) and macrophage colony stimulating factor (M-CSF) (Kaushansky, 1998). IL-6 promotes tumor angiogenesis, metastasis and metabolism (Kumari et al., 2016). Moreover, the cytokines G-CSF and M-CSF that be secreted by tumor cells could stimulate megakaryopoiesis and subsequent thrombopoiesis in cancer (Kowanetz et al., 2010). 

There is a complex interplay between platelet-induced tumor growth and tumor cell-induced platelet activation (Tesfamariam, 2016). In cervical carcinoma, platelet-derived growth factor (PDGF) receptors and their ligands are frequently expressed in cervical cancer and the majority exhibited a combination of family members co-expression (Taja-Chayeb et al., 2006). Furthermore, PDGFR antagonist imatinib blocked tumor growth (Taja-Chayeb et al., 2006). These discoveries make the PDGFR/PDGF system an attractive oncologic therapeutic target. In agreement with the studies above, our study indirectly confirmed the findings using a simple platelet index. In addition, our results indicated that evaluation of preoperative PDW levels will improve risk prediction and help physicians to better make therapeutic decisions.

Our study had several limitations. First, this was a single-center study and additional larger multicenter studies are needed to confirm our results. Second, whether our results could be generalized to other ethnic groups requires further investigation.

In conclusion, the combined use of preoperative PDW and D-dimer may be useful in predicting postoperative DVT events in patients with cervical carcinoma.

## Grant Support

This work was supported by Science Foundation of Heilongjiang Academy of Medical Science (Grant No. ZD2017017).

## References

[B1] Cui MM, Li N, Liu X (2017). Platelet distribution width correlates with prognosis of non-small cell lung cancer. Sci Rep.

[B2] Heit JA, Silverstein MD, Mohr DN (1999). Predictors of survival after deep vein thrombosis and pulmonary embolism: a population-based, cohort study. Arch Intern Med.

[B3] Kaito K, Otsubo H, Usui N (2005). Platelet size deviation width, platelet large cell ratio, and mean platelet volume have sufficient sensitivity and specificity in the diagnosis of immune thrombocytopenia. Br J Haematol.

[B4] Kaushansky K (1998). Growth factors and hematopoietic cell fate A new feature: controversies in hematology. Blood.

[B5] Kowanetz M, Wu X, Lee J (2010). Granulocyte-colony stimulating factor promotes lung metastasis through mobilization of Ly6G+Ly6C+ granulocytes. Proc Natl Acad Sci U S A.

[B6] Kumari N, Dwarakanath BS, Das A, Bhatt AN (2016). Role of interleukin-6 in cancer progression and therapeutic resistance. Tumour Biol.

[B7] Linden MD, Jackson DE (2010). Platelets: pleiotropic roles in atherogenesis and atherothrombosis. Int J Biochem Cell Biol.

[B8] Paulus JM (1981). Recent advances in the story of megakaryocyte physiology. Pathol Biol (Paris).

[B9] Schaefer JK, Jacobs B, Wakefield TW, Sood SL (2017). New biomarkers and imaging approaches for the diagnosis of deep venous thrombosis. Curr Opin Hematol.

[B10] Stender MT, Frøkjaer JB, Larsen TB (2009). Preoperative plasma D-dimer is a predictor of postoperative deep venous thrombosis in colorectal cancer patients: a clinical, prospective cohort study with one-year follow-up. Dis Colon Rectum.

[B11] Taja-Chayeb L, Chavez-Blanco A, Martínez-Tlahuel J (2006). Expression of platelet derived growth factor family members and the potential role of imatinib mesylate for cervical cancer. Cancer Cell Int.

[B12] Tesfamariam B (2016). Involvement of platelets in tumor cell metastasis. Pharmacol Ther.

[B13] Zhang H, Liu L, Fu S (2017). Higher platelet distribution width predicts poor prognosis in laryngeal cancer. Oncotarget.

[B14] Zhang X, Cui MM, Fu S (2017). Platelet distribution width correlates with prognosis of gastric cancer. Oncotarget.

